# A standardized implementation of multicenter quality improvement program of very low birth weight newborns could significantly reduce admission hypothermia and improve outcomes

**DOI:** 10.1186/s12887-022-03310-5

**Published:** 2022-05-14

**Authors:** Shu-yu Bi, Yong-hui Yu, Cong Li, Ping Xu, Hai-yan Xu, Jia-hui Li, Qiong-yu Liu, Min Li, Xin-jian Liu, Hui Wang

**Affiliations:** 1 Department of Neonatology, Shandong Provincial Hospital, Cheeloo College of Medicine, Shandong University, Jinan, 250021 Shandong China; 2 Department of Neonatology, Shandong Provincial Hospital Affiliated to Shandong First Medical University, Jinan, 250021 China; 3 Department of Neonatology, Liaocheng People’s Hospital, Liaocheng, 252000 Shandong China; 4 Department of Neonatology, The First Affiliated Hospital of Shandong First Medical University, Jinan, 250014 Shandong China; 5 Department of Neonatology, Women and Children’s Healthcare Hospital of Linyi, Linyi, 276000 Shandong China; 6 Department of Neonatology, Hebei Petro China Central Hospital, Langfang, 065000 Hebei China

**Keywords:** Very low birth weight, Neonates, Hypothermia, Quality improvement, Outcomes

## Abstract

**Background:**

Admission hypothermia (AH, < 36.5℃) remains a major challenge for global neonatal survival, especially in developing countries. Baseline research shows nearly 89.3% of very low birth weight (VLBW, < 1500 g) infants suffer from AH in China. Therefore, a prospective multicentric quality improvement (QI) initiative to reduce regional AH and improve outcomes among VLBW neonates was implemented.

**Methods:**

The study used a sequential Plan—Do—Study—Act (PDSA) approach. Clinical data were collected prospectively from 5 NICUs within the Sino-Northern Neonatal Network (SNN) in China. The hypothermia prevention bundle came into practice on January 1, 2019. The clinical characteristics and outcomes data in the pre-QI phase (January 1, 2018– December 31, 2018) were compared with that from the post-QI phase (January 1, 2019–December 31, 2020). Clinical characteristics and outcomes data were analyzed.

**Results:**

A total of 750 in-born VLBW infants were enrolled in the study, 270 in the pre-QI period and 480 in the post- QI period, respectively. There were no significant differences in clinical characteristics of infants between these two phases. Compared with pre-QI period, the incidence of AH was decreased significantly after the QI initiative implementation in the post-QI period (95.9% vs. 71.3%, *P* < 0.01). Incidence of admission moderate-to-severe hypothermia (AMSH, < 36℃) also decreased significantly, manifesting a reduction to 38.5% in the post-QI (68.5% vs 30%, *P* < 0.01). Average admission temperature improved from after QI (35.5 $\pm$ 0.7℃ vs. 36.0 $\pm$ 0.6℃, *P* < 0.01). There was no increase in proportion the number of infants with a temperature of > 37.5 °C or thermal burns between the two groups. The risk ratio of mortality in infants during the post-QI period was significantly lower in the post-QI period as compared to the pre-QI period [adjusted risk ratio (aRR): 0.26, 95% confidence interval (CI): 0.13–0.50]. The risk ratio of late-onset neonatal sepsis (LOS) also significantly lowered in the post-QI period (aRR: 0.66, 95% CI: 0.50–0.87).

**Conclusion:**

Implementation of multicentric thermoregulatory QI resulted in a significant reduction in AH and AMSH in VLBW neonates with associated reduction in mortality. We gained a lot from the QI, and successfully aroused the attention of perinatal medical staff to neonatal AH. This provided a premise for continuous quality improvement of AH in the future, and might provide a reference for implementation of similar interventions in developing countries.

**Trial registration:**

Trial registration number: ChiCTR1900020861. Date of registration: 21 January 2019(21/01/2019). Prospectively registered.

**Supplementary Information:**

The online version contains supplementary material available at 10.1186/s12887-022-03310-5.

## Background

With a large surface area to body weight ratio, thin subcutaneous fat content, poorly developed metabolic system, and inconspicuous heat consumption, very low birth weight (VLBW, < 1500 g) infants are at extremely high risk of rapid heat loss [1–3]. Admission hypothermia (AH, < 36.5℃) is common in inborn infants at hospitals (32% to 85%) and homes (11% to 92%), even in tropical environments. Thus, AH poses one of the major challenges for neonate survival globally, especially in developing countries [4–6]. AH syndromes can be accompanied by respiratory distress syndrome (RDS), intraventricular hemorrhage (IVH), and late-onset neonatal sepsis (LOS) [5, 7]. Compared with normothermic infants (36.5- 37.5 °C), the adjusted odds ratios (ORs) of all deaths have been shown to increase by 4.15 [95% confidence interval (CI): 1.51–11.44] and 1.81 (95% CI: 0.65–5.01) for infants with moderate-to-severe hypothermia (< 36℃) and mild hypothermia (36–36.4℃), respectively [5]. Since 2014, given a practical scientific idea, the concepts of prompt reflection of scientific knowledge in work, seeking help, and incorporating feedback from others in the team, have begun to be accepted by the public [8]. Several initiatives have been started to improve the quality of life of hypothermia patients involving well-characterized multifaceted heat preservation methods, such as increasing the ambient temperature, using plastic membrane, wearing a preheated wool cap, using a transportation incubator, placing chemically preheated mattress, and adjustment of the actual temperature in the perinatal medical facility to prevent hypothermia and improve the outcome. Currently, the global incidence rate of AH ranges from 10 to 34% [9–12]. An Indian study has shown significant improvement in this field by adjusting the mean admission temperature of VLBW neonates (35.3 ± 0.6 °C, 36.0 ± 0.8 °C, and 36.4 ± 0.4 °C during the pre-intervention, intervention, and post-intervention phase, respectively), which subsequently improved the composite neonatal adverse outcomes from 31% to 13.2% by hypothermia QI project [11].

However, a similar practice of hypothermia QI program has not been reported in China. In 2017, we retrospectively investigated the distribution of body temperature at admission and implementation of thermal insulation measures of VLBW neonates in 24 units in Shandong Province and found that nearly 89.3% of VLBW infants were hypothermic (< 36.5℃), and more than 50% of infants suffered from admission moderate-to-severe hypothermic (AMSH, < 36 ℃) shocks at admission to the Neonatal Intensive Care Unit (NICU). The number of NICUs that implement those important hypothermia prevention measures including the use of the plastic membrane, wrapping with a preheated wool cap, frequent monitoring and recording of body temperatures, application of transportation incubator, monthly reporting of hypothermia situation, making Plan-Do-Study-Act (PDSA) circle management strategy in 24 medical centers was lower than 50% of the total neonatal care facilities. Cold environment, lack of attention and insufficient patient management by medical staff, inadequate insulation measures, and unavailability of essential equipment were founded to be the main contributors to the high incidence of VLBW neonates with hypothermia [6, 13]. How to apply and practice these advanced hypothermia quality improvement theories and methods in detail, ensuring its efficiency in the Chinese population, are worthy of study. To optimize and standardize the thermoregulation management system, we undertook a collaborative QI initiative involving 5 provincial and regional perinatal medical facilities to improve the regional perinatal healthcare quality among VLBW neonates. Hence, we initiated a quality improvement project to reduce the incidence of AH by at least 10% over two years and to evaluate the impact of this QI project on mortality and morbidity in VLBW neonates [13].

## Methods

### Study design and settings

The high incidence of AH was noticed in the monthly reports and statistical analysis of prospectively collected data. We retrospectively investigated the temperature distribution and thermal insulation measures in 24 NICUs in 2017 and found that the number of NICUs implementing important hypothermia prevention measures, including the use of a plastic membrane, wrapping with a preheated wool cap, frequent checking of the body temperature, application of transportation incubator, monthly reporting of hypothermia situation, making Plan-Do-Study-Act (PDSA) circle management strategy in 24 neonatal facilities was lower than 50% of the total number of NICUs [6]. In subsequent studies, we identified the key drivers of AH, for example, the cold environment, lack of attention and insufficient execution paid by medical staff, inadequate insulation measures, and unavailability of essential equipment [5, 13]. By reading and learning literature and guidelines related to hypothermia QI [7, 14–17], we established an overall hypothermia quality improvement plan (Table 1, including 20 insulation measures). Considering that the implementation of 20 hypothermia preservation measures in one bundle could be challenging in a single shot, small and continuous PDSA cycles were divided and implemented in stages according to the specific implementation strategy. That guarantees the step-by-step implementation of thermal interventions.

**Table 1:** Bundle of Temperature management procedure at NICUs

Prenatal preparation
1. Prenatal preparation (prenatal consultation, form multidisciplinary team, check materials)
2.Set the ambient temperature above 25 °C, set radiant warmers at 34℃
3.Prewarm the hat
4.Prepare a polyethylene wrap
5. Push a heated transport incubator forward to delivery or operating room, plug it in and keep charging, switch on incubator and set it to target temperature range:36–36.5 °C
**Resuscitation euthermia**
6.Preheated blankets wrapping after birth
7.Quickly weight after being placed in a pre-warmed blanket
8.Infant immediately after birth wrapped with a polyethylene wrap without drying
9.Place a pre-warmed hat on the head
10.Resuscitation under chemical preheated mattress
11.Document temperature at 10 min after birth
**Transportation euthermia**
12. Put infants into a heated transport incubator and start transportation
**After admission to NICU**
13.Preheat daily materials in incubator (diapers, oxygen probe, stethoscope)
14.Put the infants into the incubator immediately when arriving at NICU
15. Document temperature continuously within one hour after birth
16. Retest temperature when arriving at the NICU, soon after every 30 min
17. Document the time point when temperature at ≥ 36.5℃
18.Nuring and medical operations are centralized implemented
19.Training and assessments on temperature measurement for nurses, making temperature measurement standard
20. Monthly charts reporting on hypothermia distribution and data quality, doing Plan–Do–Study–Act circles continuously

This prospective, multicentre cohort study was carried out over a period of 36 months, from January 1, 2018, to December 31, 2020, included five perinatal medical facilities were the level 3 NICUs and had considerable population densities. The 5 recruited hospitals that volunteered to participate in the QI initiative included 4 general hospitals and 1 maternal and child health care hospital, with an average number of 34 and 30 beds in the neonatology department and NICUs, respectively. NICUs of these hospitals were used to receive an average of 3536 newborns per year, of which approximately 123 cases (3.5%) were VLBW. The average ratios of nurses to beds and physicians to nurses ration were about 1:1 and 1: 2, respectively. All VLBW neonates admitted to the NICU were recruited to the study. Neonates who were outborn or with redirection of intensive care or congenital anomalies, missing temperature data and whose mother had a fever during delivery (temperature ≥ 38.4 °C) were excluded. We used the same digital laser infrared thermometer (OMRON, MC-347) for temperature measurements in NICUs and calibrated once a month to avoid errors. We assembled an interdisciplinary collaborative group named Hypothermia Clinical Research Group (HCRG) to develop an initial heat preservation bundle by referring to literature and guidelines. The bundles were developed based on the medical literature review, the best practice recommendations of the California Perinatal Quality Care Collaborative (CPQCC) [14], the World Health Organization (WHO), and the evidence-based principle of neonatal resuscitation projects [7, 15–17].

### Study variables

The characteristic variables were as follows: gestational age (GA), birth weight (BW), sex(boy), small for gestational age (SGA) (defined as a BW lower than the 10th percentile of the intrauterine growth curve of 2013- Fenton), multiple births (twins or more), 5-min Apgar score < 7, caesarean section, intubation in the delivery room, maternal hypertension, antenatal use of full course of steroid. Outcome variables included AH, AMSH, mortality, grade 3 or 4 IVH, grade 3 or 4 ROP, and stage 2 or higher NEC, LOS, moderate-to-severe BPD, pulmonary hemorrhage, and composite outcome (including death before discharge or any of the major morbidities: grade 3 or 4 IVH, grade 3 or 4 ROP, and stage 2 or higher NEC). In previous studies, we analyzed risk factors for AH and found that AH was associated with small GA, low BW, low Apgar score and SGA [5]. To ensure the stability of outcomes analysis, we adjusted these factors in comparing risk ratio of outcomes between two groups in the Poisson regressions.

The study was divided into two phases, including the pre-QI period (January 2018 to December 2018), and the post-QI period (January 2019 to December 2020). Interventions were confirmed on December 31, 2018, and came into use on January 1, 2019. A multidisciplinary team composed of medical and nursing staff from neonatology, obstetrics, and anesthesiology were established to implement QI practices. During the QI period, we used PDSA planning to adjust or expand interventions to decrease AH.

### The outline of sequential PDSA cycles to adjust or expand interventions during the QI phase

#### Initial Bundles (January 1–March 31, 2019)

- (1) Prenatal preparations (prenatal consultation, formation of the multidisciplinary team, checking materials),
- (2)Setting the ambient temperature: Turn on the heating mode of the air conditioner and set the temperature above 25 °C and set radiant warmers at 34℃,
- (3) Quickly dry the infant after birth,
- (4) Place a pre-warmed hat made of stockinette or wool on the head,
- (5) Weigh after being placed in a pre-warmed blanket,
- (6) Use chemically preheated mattress,
- (7) Document temperature at key time points (10 min after birth, at arrival at the NICU, soon after every 30 min, till the temperature reaches $\ge$ 36.5℃),
- (8) Training and assessments for nurses on the temperature measurement, making temperature measurement a standard procedure,
- (9) Distribution of monthly charts reporting on hypothermia and regular quality control checking.

#### PDSA Cycle 1 (April 1–May 31, 2019)

Use of polyethylene occlusive skin wraps at birth preventing heat loss.

### PDSA Cycle 2 (June 1–August 31, 2019)

Introduction of a preheated portable incubator to keep the infant warm during transportation

### PDSA Cycle 3 (September 1,2019 – March 31, 2020)

Revision of AH checklist- adding individual signature in the blank area on the checklist to supervise effectively, and feedback checklist would be completed at weekly meetings.

### PDSA Cycle 4 (April 1– December 31, 2020)

Providing various online education lectures to medical staff and further emphasizing the warmth link in the stabilization of the “golden hour” for VLBW infants in NICUs by monthly online literature sharing and learning.

The 4 cycles cover different links in the process of keeping the newborn warm, namely resuscitation, transportation, handover, and shared learning. In the process of resuscitation, especially for premature babies with small gestational age or lower weight, the method of using plastic wrap to keep warm can better prevent the loss of water and heat through evaporation, radiation, and convection. In addition to being economical, it can avoid the discomfort caused by the roughness of the repeatedly sterilized towels used for wrapping newborns. Therefore, to improve the warming procedure, we replaced the traditional method of wiping dry immediately after birth with the polyethylene membrane with 30 cm × 40 cm size to wrap babies, which could allow infants’ head, torso, and limbs to be covered totally for better insulation [18–20].

In regular feedback and interviews, we found significant differences between the NICUs regarding transport warmth. Most units said they did not focus on the special transport warmth requirement. Instead, they simply wrapped infants before taking them to the NICU. Transport distance might vary in the actual situation depending on the locations of the delivery room or operating room across NICUs, for example, some could be on different floors of the same building, others could be even in different buildings. To solve this problem, we introduced an insulated pre-heated portable incubator for transportation in June 2019 [20, 21], adding the pre-transport preheating process in the delivery room or operating room in a bundle.

We found that the compliance of the bundle declined in September 2019. The identified reasons from onsite visits were insufficient staffing and inadequate force of supervision in handover. With a relatively tense doctor-patient ratio in China, reducing turnover in staff is not feasible. To ensure the efficiency and feasibility of measures, the executive chairman of the Sino-Northern Neonatal Network (SNN) unit suggested revising the debriefing AH worklist for VLBW infants, adding signature blank to individual responsibility, and getting weekly feedback according to local conditions in the respective unit. The changed version was tried on a small scale for 1 week for improved compliance and was further promoted to all 5 perinatal medical centers. The revised paper version of the worklist was listed as a necessary item. A list of necessary prenatal items was pasted on the side of the rescue box as a warning to remind the pediatric consultation doctor to record the temperature in the delivery room on time. After being transferred to the NICU, the consultation staff should fill in the general information and check used thermal measures buttons in the worklist and then hand it over to the nurse on duty to complete the continuous measurement and recording task. Once the worklist was completed, it was clamped under the transparent plastic nurse workboard. On the next day, the paper was retaken by the resident doctor, and feedback was reported in every weekly meeting. The worklist for data collection was shown as in supplemental information.

During the fourth PDSA cycle in April 2020, the confounding social factors were social panic caused by the epidemic situation, reduction of salary leading to the decrease of staff's work enthusiasm due to the prevalence of the novel Coronavirus epidemic. To rebuild awareness of hypothermia in medical staff, the executive leader in SNN carried out various online education lectures to boost the confidence level and firm the faith, further emphasizing the warmth link in the stabilization of golden hour for VLBW infants by monthly online literature sharing, learning, and feedback meetings. In addition to stressing the compliance measures, the meeting also illustrated the cooperation between obstetrics and pediatrics for prenatal communication, the clusters of management of medical operations, the standardized management of processes, and the timeliness of feedback reporting. We have added a link for a prenatal consultation. Intrauterine consultation was carried out before delivery to ensure full communication between obstetrics and pediatrics consultants. On the day of delivery, the consultation form was placed half an hour in advance, and NICU was called at the same time to allow sufficient time to reserve a transport incubator.

All problems, suggestions, and temperature measurement videos were sent out to all participating centers by e-mail or WeChat discussion. The bundle emphasized the accurate documentation of temperatures at each time point. Overall specific interventions are listed in Table 1. Monthly random onsite visits by executive leaders were arranged for face-to-face communication to overcome understanding barriers and improve the supervision of data quality control. Regular in-service education and online lectures of videos on heat preservation methods were sent to the public mailbox, which could be easily accessed by pediatric and obstetric providers and nurses to improve their awareness and efficacies in correctly documenting temperatures.

### Definitions

Hypothermia was defined as a rectal temperature of less than 36.5 °C, according to the WHO [7]. Cold stress or mild hypothermia was defined as a temperature 36.0 °C to 36.4 °C; moderate hypothermia was defined as a temperature 32.0 °C to 35.9 °C, and severe hypothermia was defined as a temperature below 32 °C. Normothermia was defined as a body temperature between 36.5 °C and 37.5 °C. Redirection of intensive care was defined as the limited care (not intensifying medical treatment) or withdrawal of care [22]. The diagnostic criteria of RDS, IVH, NEC and ROP were set according to the Practice of Neonatology [23]. The composite outcome included death before discharge or any of the major morbidities including grade 3 or 4 IVH, grade 3 or 4 ROP, and stage 2 or higher NEC [11]. LOS was diagnosed by the clinical manifestations of systemic infection after 3 days of birth and abnormal values for 2 or more of the following non-specific infection indicators: WBC < 5 × ${10}^{9}$ /L or WBC > 20 × ${10}^{9}$/L; C-reactive protein (CRP) ≥ 10 mg/L; platelets (PLTs) ≤ 100 × ${10}^{9}$ /L; and procalcitonin (PCT) > 2 ng/ml [24]. Moderate-to-severe BPD was defined as the requirement of any inspired fraction of oxygen above 0.21 at the corrected GA of 36 weeks [25]. If any fresh blood appeared in the tracheal intubation, with hematocrit dropping by more than 10% in the routine blood examination, the decrease of transmittance on the chest X-ray was diagnosed as pulmonary hemorrhage [26].

### Data extraction

The admission temperature-related data of VLBW infants were collected prospectively at the SNN by a standardized operating procedure. The database provided information on maternal health, delivery details, neonatal clinical information, and temperature data before discharge [5, 13, 27]. The admission temperature was defined as the infant’s rectal temperature measured at admission to the NICU within 1 h of birth, because it’s closer to the core temperature [28]. A worklist of temperature evaluation for VLBW infants was used to carry on hand by the consulting physician before every intrapartum consultation to data collection, which documenting interventions on the back side and temperature data at different time points after birth on the front side in the supplemental information.

### Statistical analysis

Demographic data are expressed as medians [M (Q1, Q3)], mean $\pm$ standard deviation (SD), or percentages. Comparisons of clinical characteristics between the two groups were calculated with the Mann–Whitney U-test, independent-samples t-test, and chi-square (χ^2^) test. Risks of outcomes compared between the groups were tested on Poisson regression analysis. P < 0.05 was considered statistically significant. The statistical analyses were conducted using SPSS v.26.0 (SPSS Inc., Chicago, Illinois), and QI Macros 2018–09 (Denver, CO). Special cause signals were identified by using standard control chart rules [29]. This study was reported according to the framework of SQUIRE2.0 guidelines [30].

## Results

**Fig. 1:**
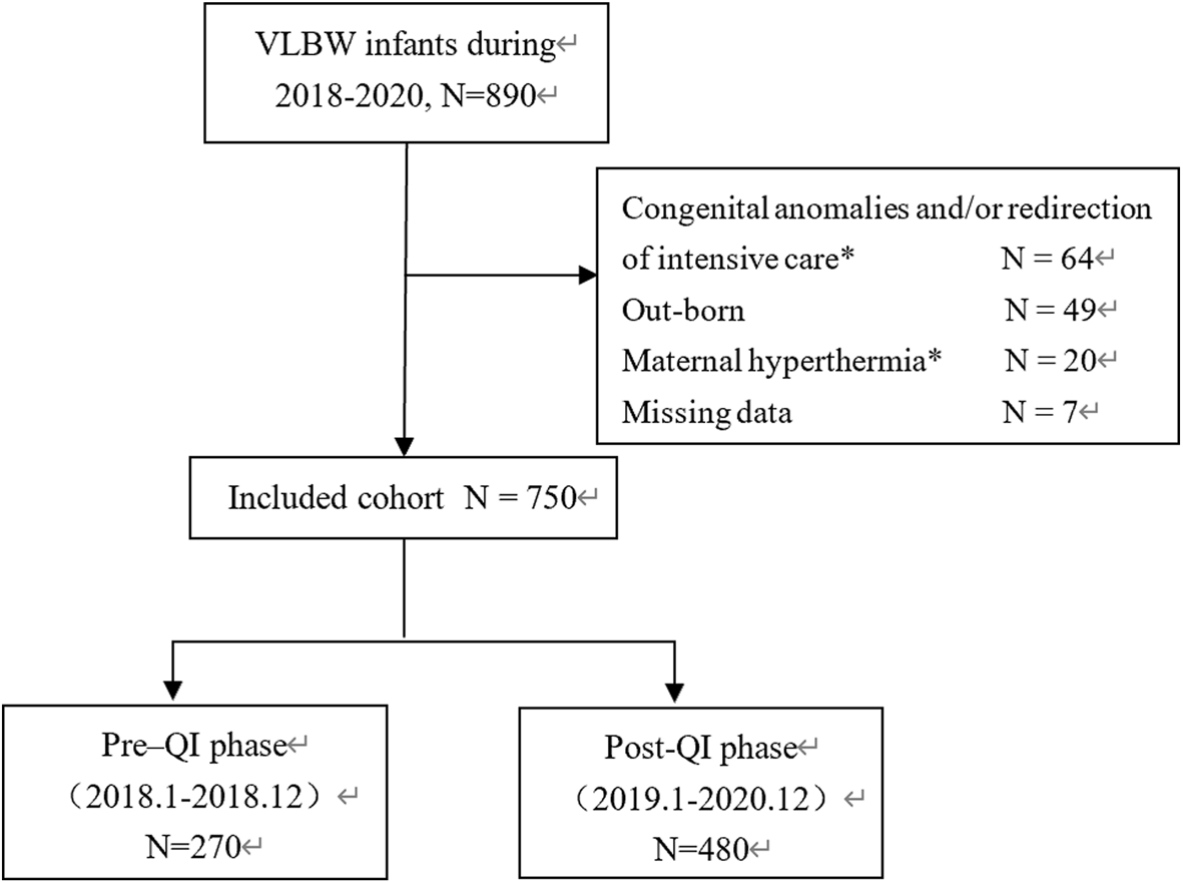
**Patient Inclusion**. A total of 890 in-born infants with a BW < 1500 g were enrolled in the study; 49 infants were excluded because they were out-born; 20 infants were excluded because their mother had a fever during delivery (temperature ≥ 38.4 °C). Additionally, 64infants with redirection of intensive care and 7 infants with missing temperature data were excluded. The remaining 750 VLBWIs were included in this analysis, 270 infants in pre-QI phase and 480 infants in post-QI phase, respectively. (*: limited care (not intensifying medical treatment) or withdrawal of care; Maternal hyperthermia*: mothers had a fever temperature (≥ 38.4 °C) during delivery)

During the study period, 890 neonates were admitted, of which 750 VLBW neonates were included in this study. Two hundred seventy neonates in the pre-QI period and 480 in the post-QI period, respectively (Fig. 1). Infants born during both periods had no significant differences in neonatal and perinatal clinical characteristics, including GA, gender, birth weight, multiple gestations, cesarean section, the percentage of neonates with 5-min Apgar score < 7, and so on (Table 2).

**Table 2:** Characteristics of VLBW infants in pre-QI and post-QI group

	Pre-QI phase (*n* = 270)	Post-QI phase (*n* = 480)	P^*^
GA [weeks, Medians (Q1, Q3)]	29.6 (28.3, 31.4)	29.7 (28.3, 31.0)	0.954
GA < 28 weeks, (%)	47 (17.4)	98 (20.4)	0.316
BW [g, Medians (Q1, Q3)]	1210 (1000, 1360)	1200 (1000, 1368)	0.937
BW < 1000 g, n (%)	58 (21.5)	117 (24.4)	0.368
Sex (boy), n (%)	150 (55.6)	233 (48.5)	0.065
SGA, n (%)	40 (14.8)	64 (13.3)	0.573
Caesarean section, n (%)	221 (81.9)	373 (78.5)	0.278
Multiple gestation (twins or more), n (%)	57 (21.1)	113 (23.5)	0.445
Apgar score at 5 min < 7, n (%)	40 (14.8)	56 (11.7)	0.219
Intubation at delivery room, n (%)	80 (29.6)	135 (28.1)	0.662
Maternal hypertension, n (%)	124 (45.9)	182 (39.9)	0.113
Antenatal use of full course of steroid, n (%)	163 (64.2)	273 (67.2)	0.418

Data are presented as medians [Medians (Q1, Q3)] or n (%)

Abbreviations: *QI* Quality improvement, *GA* Gestational age, *BW* Birth weight, *SGA* Small for gestational age

^*^ Mann–Whitney U-test or Chi-Square test

### Hypothermia

**Fig. 2:**
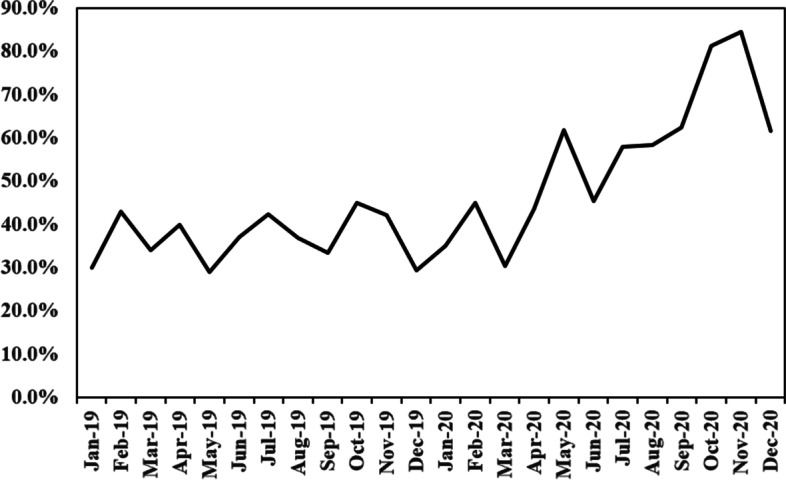
Month proportion of rectal temperature ≥ 36.5℃ within first hour after admission during the QI phase

**Fig. 3:**
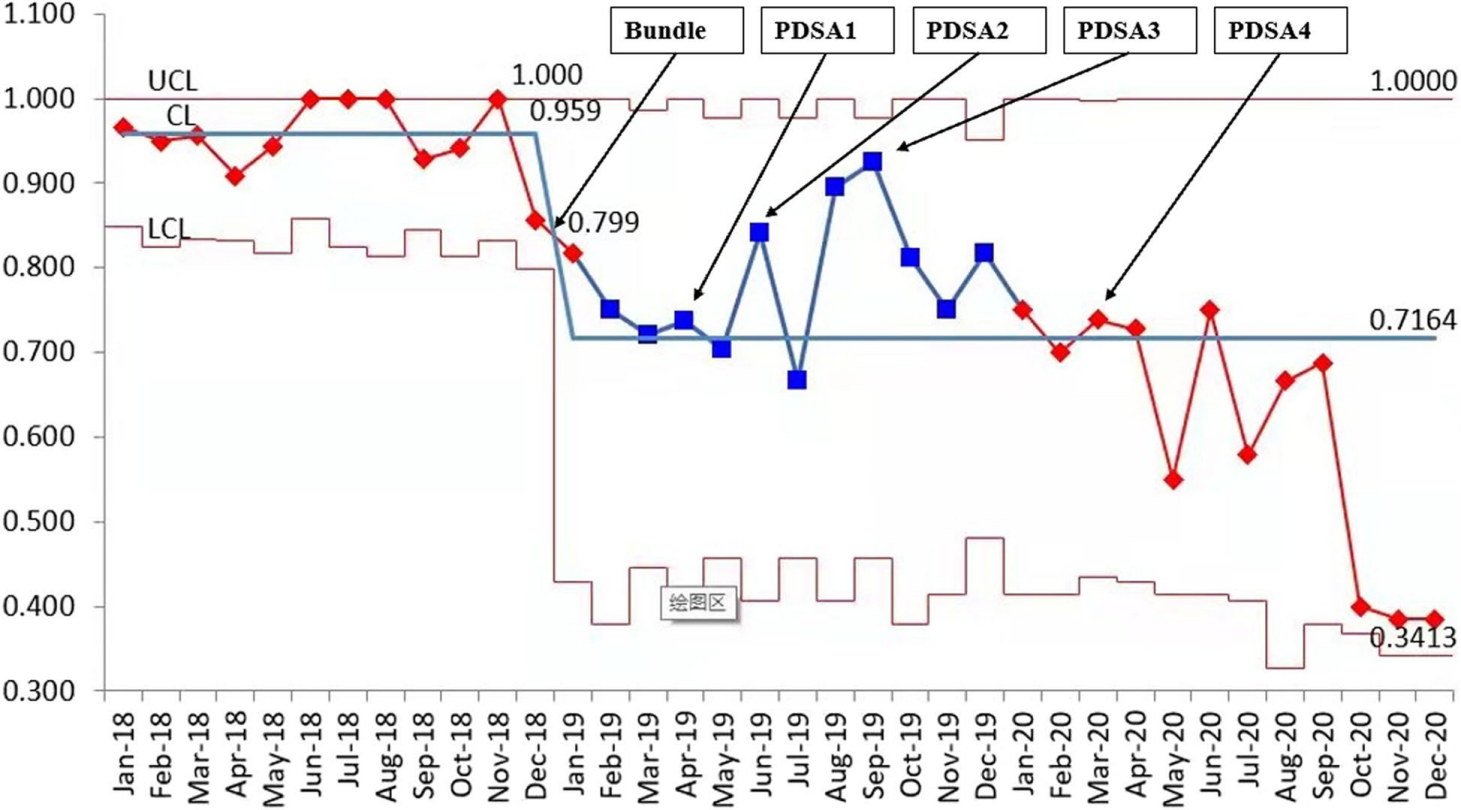
P-chart of monthly AH percentage in January 2018–December 2020. Subdivided into pre-QI period and post-QI period. CL, center line; LCL, lower control limit; UCL, upper control limit. Arrows show change of major interventions including the thermoregulation bundle: *Initial Bundles (January 1–March 31, 2019); PDSA Cycle 1 (April 1–May 31, 2019):* Using polyethylene occlusive wrap infants without drying instead of drying infants immediately after birth. *PDSA Cycle 2 (June 1–August 31, 2019):* a heated transport incubator introduction. *PDSA Cycle 3 (September 1,2019 – March 31, 2020):* Revise admission hypothermia check list to supervise effectively. *PDSA Cycle 4 (April 1– December 31, 2020):* Various online education lectures monthly to build up confidence, faith and further emphasize heat preservation awareness

Table 3 shows the distribution of AH in the different periods of QI. The number of infants with AH at PDSA cycles in the post-QI period all showed different degrees of decline comparing with the baseline data in the pre-QI period. Compared with the pre-QI period, the percentage of VLBW infants with AH was decreased in the QI period (95.9% vs. 71.3%, *P* < 0.01). AMSH was improved significantly by reducing to 38.5% after QI (68.5% vs. 30%, *P* < 0.01). The proportion of neonates with normothermia gained an increase of by 24.2% after QI (4.1% vs. 28.3%, *P* < 0.01). Average admission temperature improved after QI (35.5 $\pm$ 0.7 vs. 36.0 $\pm$ 0.6, *P* < 0.01). There was no significant increase in the rate of hyperthermia (0.4% vs. 0%). No thermal burns were reported. The proportion of rectal temperature was ≥ 36.5℃ within the first hour of admission when the QI phase was gradually decreased over time (Fig. 2). The risk of infants with AH was lower in the post-QI period as compared to the pre-QI period [adjusted risk ratio(aRR): 0.74, 95% CI: 0.63–0.87]. The risks of infants with AMSH were also decreased significantly in the post-QI phase (aRR: 0.44, 95% CI: 0.35–0.54) **(Table **5). The control P chart revealed that the central line was shifting down from a baseline of 95.9% to 71.6% during the post-QI phase, which was consistent with the temperature distribution (Fig. 3). The improvement was still ongoing in the chart.

**Table 3:** Admission temperature distribution among VLBW infants in pre-QI, and post- QI phases

	Pre-QI phase	Post-QI phase						P*
	(*N* = 270)	Initial bundle (2019.1–3) *n* = 61	PDSA1 (2019.4–5) *n* = 46	PDSA2 (2019.6–8) *n* = 64	PDSA3 (2019.9–2020.3) *n* = 159	PDSA4 (2020.4–12) *n* = 150	ALL *N* = 480	
Temperature, mean $\pm$ SD	35.5 $\pm$ 0.7	36.1 $\pm$ 0.7	36.2 $\pm$ 0.5	36.0 $\pm$ 0.7	35.9 $\pm$ 0.7	36.2 $\pm$ 0.6	36.0 $\pm$ 0.6	< 0.001
AH, n (%)	259(95.9)	46(75..4)	32(69.6)	50(78.1)	126(79.2)	88(58.6)	342(71.3)	< 0.001
AMSH, n (%)	185(68.5)	9(14.8)	10(21.7)	19(29.7)	68(42.8)	38(25.3)	144(30.0)	< 0.001
Normothermia, n (%)	11(4.1)	14(23.0)	14(30.4)	14(21.9)	32(20.1)	61(40.7)	136(28.3)	< 0.001
Hyperthermia, n (%)	0(0.0)	1(1.6)	0(0.0)	0(0.0)	0(0.0)	1(0.7)	2(0.4)	0.288

Data are presented as the mean $\pm$ SD or n (%); Abbreviations: *QI* Quality improvement, *PDSA* Plan-do -study-act, *SD* standard deviations, *AH* Admission temperature, < 36.5℃; AMSH Admission moderate/severe temperature, < 36℃

^*^ Independent-samples T test or Chi-Square test for pre-QI(*n* = 270) and post-QI phases(*n* = 480) comparison

### Outcomes

Compared with the pre-QI phase, the mortality rate of VLBW neonates was declined significantly from 10.7% to 2.7% (*P* < 0.01) in the post-QI phase (Table 4). The incidence of LOS was also reduced by 11% in the post-QI phase, from 33 to 22% (*P* < 0.01) (Table 4). Compared to the pre-QI period, the unadjusted RR of mortality and LOS for the post-QI period were separately reduced to 0.25 (95% CI: 0.13–0.48) and 0.66 (95% CI: 0.52–0.84) (Table 5). The risk ratios of mortality and LOS remained significant lower, respectively, after adjusting for the confounding factors such as BW, GA, SGA, and Apgar scores at 5 min < 7 (aRR: 0.26, 95% CI: 0.13–0.50; aRR: 0.66, 95% CI: 0.50–0.87) (Table 5). No significant difference was observed between the two groups on the composite outcome, incidences of NEC (≥ stage 2), BPD (≥ stage 2), IVH (≥ grade 3), and ROP (≥ grade 3) (Table 5).

**Table 4:** Comparison of adverse outcomes among VLBW neonates in pre-QI and post- QI phase

Outcome	Pre-QI phase (*n* = 270)	Post-QI phase (*n* = 480)	P ^a^
Composite outcome, n (%)	101 (37.4)	159 (33.1)	0.237
Mortality, n (%)	29 (10.7)	13 (2.7)	< 0.001
LOS, n (%)	89 (33.0)	105 (21.9)	0.001
NEC (Bell stage ≥ 2), n (%)	14 (5.2)	13 (2.7)	0.081
IVH (Papile grade 3/4), n (%)	6 (2.2)	17 (3.5)	0.314
Pulmonary hemorrhage, n (%)	17 (6.3)	18 (3.8)	0.113
BPD (moderate/severe), n (%)	27 (10.0)	53 (11.0)	0.657
ROP (grade 3/4), n (%)	3 (1.1)	7 (1.7)	0.754

Data are presented as n (%). Abbreviations: *QI* Quality improvement, *IVH*, Intraventricular haemorrhage, *NEC* Necrotizing enterocolitis, *LOS* Late-onset neonatal sepsis, *BPD* Bronchopulmonary dysplasia, *ROP* Retinopathy of prematurity

Composite outcome included death before discharge or any of major morbidity including grade 3 or 4 IVH, grade 3 or 4 ROP, moderate/severe BPD and stage 2 or higher NEC

^a^ Chi-Square test

**Table 5:** Unadjusted and adjusted relative risk of outcomes for VLBW infants during post-QI phase with reference to pre-QI phase

Adverse outcomes	Pre-QI phase (*n* = 270)	Post-QI phase (*n* = 480)	
		Unadjusted RR (95% CI)	^b^Adjusted RR (95% CI)
Hypothermia	1.00	0.74 (0.70, 0.79)	0.74 (0.63, 0.87)
Moderate/severe hypothermia	1.00	0.44 (0.37, 0.51)	0.44 (0.35, 0.54)
Composite outcome	1.00	0.89 (0.73, 1.08)	0.89 (0.69, 1.14)
Mortality	1.00	0.25 (0.13, 0.48)	0.26 (0.13, 0.50)
LOS	1.00	0.66 (0.52, 0.84)	0.66 (0.50, 0.87)
NEC (Bell stage ≥ 2)	1.00	0.52 (0.25, 1.10)	0.54 (0.25, 1.15)
IVH or PVL (Papile grade 3/4)	1.00	1.60 (0.64, 4.00)	1.77 (0.69, 4.51)
Pulmonary hemorrhage	1.00	0.60 (0.31, 1.14)	0.61 (0.32, 1.20)
BPD (moderate/severe)	1.00	1.10 (0.71, 1.71)	1.11 (0.70, 1.78)
ROP (grade 3/4)	1.00	1.50 (0.40, 5.62)	1.49 (0.39, 5.62)

Abbreviations: *QI* Quality improvement, *RR* Risk ratio, *CI* Confidence interval, *BPD* Bronchopulmonary dysplasia, *IVH* Intraventricular haemorrhage, *NEC* Necrotizing enterocolitis, *LOS* Late-onset neonatal sepsis, *ROP* Retinopathy of prematurity, *GA* Gestational age, *BW* Birth weight, *SGA* Small for gestational age

^b^ Adjusted for BW, GA, SGA, Apgar score at 5 min < 7 by Poisson regression analysis

## Discussion

With such a high incidence of hypothermia in baseline, it is surprising that substantial improvement could be achieved in the reduction of AH and neonatal outcomes with the initial implementation of a multicener regional quality improvement program. In this study, we showed a substantial reduction in incidences of VLBW infants with AH by at least 10% over two years. The implementation of the hypothermia bundle using PDSA methodology led to a decrease in the prevalence of hypothermia, from 95.9% to 71.3%. Moderate-to-severe hypothermia was also declined significantly, from 68.5% to 30%. The risk of hyperthermia did not increase. The risk ratio of mortality and LOS were lower individually declined to at 0.26 (95% CI: 0.13–0.50) and 0.66(95% CI 0.50–0.87), respectively. By standardizing the heat preservation management procedure, awareness of hypothermia among staff could be emphasized to some extent, which accidentally made it an invisible intervention to contribute to ongoing improvement.

With unbalanced medical resources and a large gap between urban and rural areas, hypothermia in developing countries is still a serious situation and a major challenge to newborn survival worldwide [4]. Studies show that AH is an independent risk factor for neonatal mortality and morbidity, associated with a high likelihood of IVH and LOS [5, 31, 32]. Recognizing the current state of hypothermia in neonatal care units is conducive to sound the alarm to medical personnel, prompting them to make efforts to find the cause, strengthen awareness of the disease, contribute to implementing targeted clinical quality improvement, enhance the quality of care and improve the clinical neonatal outcomes [9–12, 18, 33].

The main advantage of this study is that it objectively shows a successful practice of the targeted multicentric AH quality improvement for VLBW infants in northern China. Prospective and substantial multicentric samples in research provide the credibility of reliable results. Moreover, it contributes to the global epidemiology of hypothermia, indicating that hypothermia remains a major challenge in developing countries, especially in China. Improvement in the action and awareness are key parts to promote a paradigm shift in AH management [7, 9–12, 18, 33]. Most importantly, this study provides an instant method to figure out problematic issues and find solutions, which may promote continuous quality improvement. The supervision and training of employees increase the possibility of standardized temperature measurement, it can improve accuracy to the greatest extent and avoid bias. In our study, random spot visits facilitated the supervision of each unit and contributed to the timely identification of the obstacles encountered in the improvement process.

Studies have shown that the implementation of hypothermia quality improvement projects can reduce hypothermia-related deaths and complications and improve prognosis [9, 11, 12, 33]. A single-centered QI study from India for 6 months showed the overall reduction of AH to 37%, while the moderate hypothermia was declined from 46% to < 10% (*P* < 0.001) with a significant reduction in incidences of IVH (13% vs. 4.7%), LOS (38% vs. 19%), and metabolic acidosis (43% vs. 28%) [33]. Another QI research from Singapore reported that incidences of AH decreased from 79.4 to 40.5% (*P* < 0.001), constituting 49% of improvement (OR = 0.18, 95% CI: 0.10–0.32), though IVH and mortality rate remained unchanged [34]. In our study, the mortality and incidence of LOS in VLBW neonates were declined obviously in the post-QI phase, which was consistent with previous studies. We found no significant difference in the Characteristics of the infants in the two groups, including the perinatal and neonatal variables, and there was no significant change in the diagnosis and treatment of the complications within 3 years. The clear clinical effect after QI was obvious in the communications between units and unit of sharing experience reports, and we found more small gestational age and low birth weight infants surviving through multicenter hypothermia QI, so we think that the observed results are relevant to the intervention. We did not find any difference in the composite outcome, especially in the case of IVH (≥ grade 3) in the two groups. On the one hand, the results might differ due to different external environmental conditions, geographical locations, climatic factors, and ethnicity of the studies. Furthermore, samples in these studies might not be large, which could cause biased accuracy from sampling error. Nevertheless, the improvement is still ongoing, and the effect may be apparent in the continuous QI. Because of the lack of synergistic effect of single-center research, the findings of this multicentric study might not be comparable with previous single-centered studies.

We did not quantify the materialistic cost of the thermoregulatory bundle since its economic advantages and applicability were visible, for example, polyethylene wrapping was much cheaper than other medical-grade bags [19–21, 35]. The cost of a thermometer is US $15, which is uniformly purchased by the collaboration group. Each unit distributes 6–8 pieces per year, with a total cost of about US $90–120. Warm items such as hats, mattresses, and clothing are routine insulation items in the hospital, and the transfer incubator could be a movable inpatient bed incubator in NICUs and no additional purchases are required. All the interventions could be conducted easily and at a low cost, allowing it to refer, copy, and apply. Neither a lower Apgar score nor a more intensive recovery was observed before and after QI, indicating that there was no significant change in the opportunity cost.

How to maximize the effectiveness of each intervention depends on the good compliance, execution, and feedback of thermal insulation measures [7, 11, 12, 18–22, 35]. In our study, we paid more attention to the consistency and completeness of the implementation of measures. In QI phase, the filling rate of temperature measurement data was 97.7% (469/480), and 11 cases of missing data may be related to the fact that the staff were not able to carry the thermometer or forgot to measure in a hurry to resuscitation. The measure filling rate was 99.1% (476/480), with 4 cases of missing data. We monitored the compliance of general measures to be implemented every month and found that the average compliance of general measures in 2019 was 68.0%. The trend is more stable in 2020, with a large-scale improvement, which was 92.0%. Monthly online lecture learning, experience sharing and literature reading promoted the perinatal medical staff to establish the awareness of prevention of neonatal hypothermia and cultivated them to develop good clinical scientific thinking, and facilitated the cooperation and healthy competition (learning and reference from model units) among NICUs, at the same time, enhancing quality (compliance of process) can increase the value of interventions without increasing costs, these might be unexpected benefits in QI process.

The conversation between the executive chairman and NICU department directors during onsite visits showed that the biggest obstacle was that staff did not want to break out of their comfort zones (systematic clinical habits or conventions) to make changes. In addition to the daily tedious work, it was difficult for medical staff to act with courage with unrewarded energy and physical labor to increase the workload. Maybe this deep-rooted mentality is one of the huge difficulties for future jobs, which is of primary importance for the next plan to ensure the continuous improvement of the effect. Potential reasons for occasional residual severe hypothermia events might be related to multiple births in the operating room, prolonged asphyxiation and resuscitation, and insufficiency of preparation for emergency delivery, which further reminded us to pay more attention to these areas in the next phase.

A possible limitation of this study was the difference in results between NICUs. We standardized the temperature measurement methods and instruments, conducted continuous temperature detection and feedback recording regularly. However, variation in execution capability of thermal measures in NICUs likely contributed to different center outcomes. We didn’t evaluate those changes. Another limitation was that the total number of observation cases was decreased in 2020 (about two-thirds of the cases in 2019), which might affect our interpretation of the results, but we focused on comparing the general AH proportion between groups so that the results could have certain representativeness. Areas for future review should include strengthening the supervision in compliance with interventions in NICUs, developing leadership training for academic leaders in units and regular implementing the experience sharing and practical experience of model units to maximize the autonomy and increase the motivation of each NICU.

In developing countries, the consciousness of keeping warm is very important. We expected our initial exploration of hypothermia quality improvement to give inspiration and lessons to units or readers who were suffering from a similar condition. Although there were still shortcomings, we had gained a lot of knowledge in the QI process, like constant intercommunication, learning, and sharing, which helped achieve our goal to reduce AH in the end.

## Conclusions

Implementation of multicentric thermoregulatory QI resulted in a significant reduction in AH and AMSH in VLBW neonates with associated reduction in mortality. We learned how to identify problems in data, made targeted changes, shared experiences between NICUs, and prompted continuous multicenter QI work. Besides, we successfully aroused the attention of perinatal medical staff to AH in the region and motivated medical staff to make changes, which might be a reference to a certain extent.

## Supplementary Information

**Additional file1:** 

## Data Availability

The data that support the findings of this study are available from the corresponding authors upon reasonable request.

## Abbreviations

VLBW: Very low-birth weight
QI: Quality improvement
PDSA: Plan-do-study-act
NICU: Neonatal intensive care unit
AH: Admission hypothermia
AMSH: Admission moderate-to-severe hypothermia
HCRG: Hypothermia Clinical Research Group
CPQCC: California Perinatal Quality Care Collaborative
IQR: Interquartile ranges
GA: Gestational age
BW: Birth weight
SGA: Small for gestational age
ORs: Odds ratios
IVH: Intraventricular hemorrhage
NEC: Necrotizing enterocolitis
LOS: Late-onset neonatal sepsis
BPD: Bronchopulmonary dysplasia
ROP: Retinopathy of prematurity
SNN: Sino-Northern Neonatal Network
