# Supplementary figures and images for: A standardized implementation of multicenter quality improvement program of very low birth weight newborns could significantly reduce admission hypothermia and improve outcomes
Source: BMC Pediatr. 2022 May 14;22:281. doi: 10.1186/s12887-022-03310-5 (PMC9107002; doi:10.1186/s12887-022-03310-5)

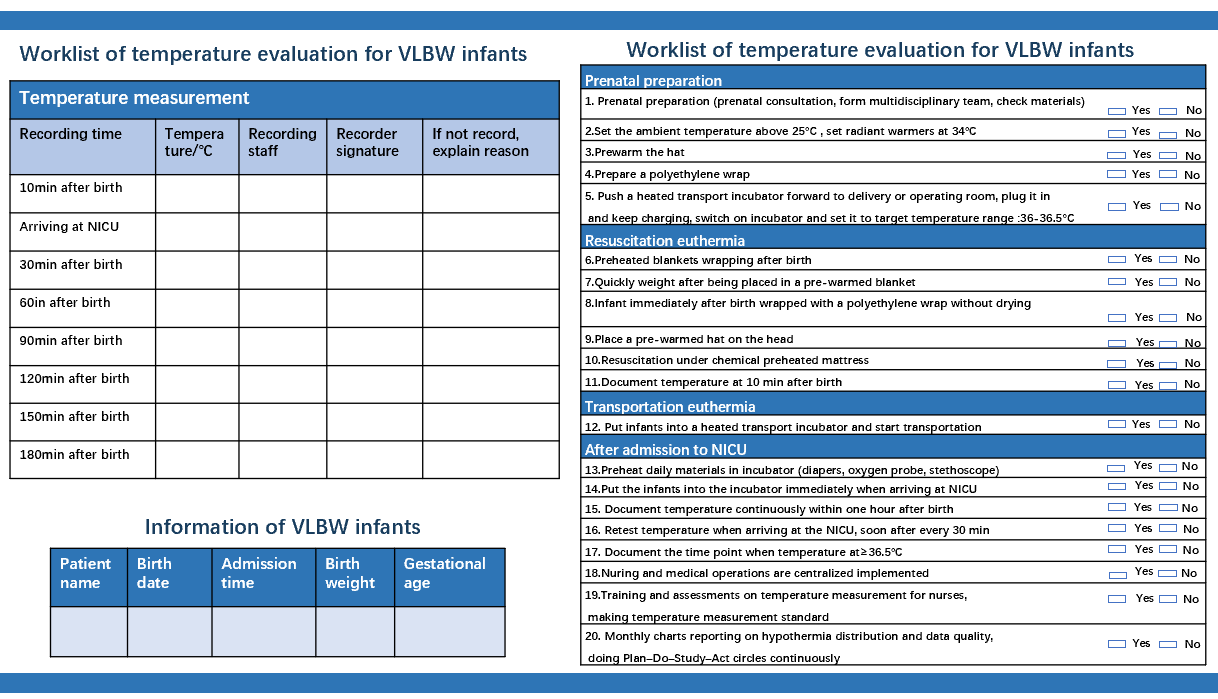

Supplement: Supplementary file 1 — Additional file1: [file 12887_2022_3310_MOESM1_ESM.bmp]
